# Bovine respiratory microbiota of feedlot cattle and its association with disease

**DOI:** 10.1186/s13567-021-01020-x

**Published:** 2022-01-12

**Authors:** Jianmin Chai, Sarah F. Capik, Beth Kegley, John T. Richeson, Jeremy G. Powell, Jiangchao Zhao

**Affiliations:** 1 Division of Agriculture, Department of Animal Science, University of Arkansas, Fayetteville, AR 72701 USA; 2 Texas A&M AgriLife Research and Department of Veterinary Pathobiology, Texas A&M College of Veterinary Medicine and Biomedical Sciences, Canyon, TX 79015 USA; 3 Department of Agricultural Sciences, West Texas A&M University, Canyon, TX 79016 USA

**Keywords:** cattle, bovine respiratory disease, microbiota, biogeography, host-microbial interaction, pneumonia

## Abstract

Bovine respiratory disease (BRD), as one of the most common and costly diseases in the beef cattle industry, has significant adverse impacts on global food security and the economic stability of the industry. The bovine respiratory microbiome is strongly associated with health and disease and may provide insights for alternative therapy when treating BRD. The niche-specific microbiome communities that colonize the inter-surface of the upper and the lower respiratory tract consist of a dynamic and complex ecological system. The correlation between the disequilibrium in the respiratory ecosystem and BRD has become a hot research topic. Hence, we summarize the pathogenesis and clinical signs of BRD and the alteration of the respiratory microbiota. Current research techniques and the biogeography of the microbiome in the healthy respiratory tract are also reviewed. We discuss the process of resident microbiota and pathogen colonization as well as the host immune response. Although associations between the microbiota and BRD have been revealed to some extent, interpreting the development of BRD in relation to respiratory microbial dysbiosis will likely be the direction for upcoming studies, which will allow us to better understand the importance of the airway microbiome and its contributions to animal health and performance.

## Introduction to the bovine respiratory microbiome

Bovine respiratory disease (BRD), a leading cause of morbidity, mortality and economic cost, is one of the largest health challenges facing the modern-day beef cattle industry [1]. In the US, over 90% of large feedlots reported BRD as the most frequent disease [2]. Not only does this disease result in increased medication costs and death, but morbid beef cattle also grow slower, develop less efficient feed conversion ratios, and tend to need additional feed time to reach similar carcass quality of clinically healthy calves [3]. The wide use of vaccines and antimicrobials to prevent and treat BRD is a common approach worldwide [2]. However, the desired effects of vaccines to protect against BRD have not been reached, and mass administration of antimicrobials should be critically evaluated due to increased concerns over antibiotic resistance [4–8]. Alternative therapies, such as probiotics [9], are becoming increasingly investigated to treat BRD and improve management. For example, intranasal bacterial therapeutics developed from the bovine nasopharyngeal *Lactobacillus spp.* could reduce the colonization by pathogen *Mannheimia haemolytica* in dairy calves [10].

In past decades, next-generation sequencing (NGS) technology has contributed to the progressive understanding of the roles of the resident microbiota [11]. The microbiome, including both the community of the microbiota (microorganisms containing bacteria, archaea, fungi, protists and algae) and their “theatre of activity” (structural elements, metabolites/signal molecules, and the surrounding environmental conditions) in a specific environment (e.g., gut, lung), are important for animal health and disease [12, 13]. The contribution of the respiratory microbiota to maintaining health and its association with disease has attracted more attention [14–16]. It is well known that, in humans, the airway microbial system may cooperate with host immunity and metabolize products to generate key defenses against infections produced by opportunistic pathogens [17, 18]. Moreover, airway microbial composition and heightened respiratory pathogen incidence are associated with pneumonia in beef cattle [19, 20]. The respiratory ecosystem contains the upper (URT) and the lower (LRT) respiratory tract at the anatomical and physiological perspectives. Regarding the specific environments of each niche in the respiratory tract, niche-associated microbiota inhabit and differentiate within each specific environment [11]. Despite the remarkable progress made in recent studies using NGS, current research relevant to the bovine respiratory microbiome is only at an initial stage. The most commonly implicated bacterial pathogens in BRD cases, including *Mycoplasma bovis*, *Histophilus somni*, *Mannheimia haemolytica* and *Pasteurella multocida*, have traditionally been identified using culture-dependent approaches [1, 21]. However, these bacteria have been classified from both clinically healthy controls and morbid cattle. Although some studies have attempted to investigate the URT and lung microbiomes using NGS [1, 20, 22–25], the microbial movements within the respiratory tract are still unknown. Based on the results in previous studies, the notion that the respiratory microbiome is significantly important to cattle health has been confirmed [15, 20, 22, 26]. Since BRD results in massive economic losses and beef cattle are one of the key food sources of human society, additional research that elucidates the role of the respiratory microbiota in BRD pathophysiology is needed and may help us identify potential alternative therapies.

In this review, we summarize BRD clinical signs and pathogenesis, techniques applied in the respiratory microbiome analysis, the biogeography of the microbiota in the respiratory system, and the association between BRD and the microbiome, which provide implications of the respiratory microbiome in health, disease and animal production for further studies. Although there are many debates regarding the respiratory microbiome, the speculation and authors’ own opinions are presented. Notably, as the host-microbiota’s interactions and microbial drifts within the airway are contemporary hot topics and will continue to be highly relevant to future studies, it is deeply analyzed in this discussion.

## The pathogenesis of bovine respiratory disease

BRD, also known as “shipping fever”, is the most frequent and costly disease of the modern beef cattle industry, especially for newly feedlot calves [6]. Recently weaned and transported beef cattle are at an even greater risk for developing BRD, which contributes to roughly 70–80% of feedlot total morbidity and roughly 10–50% of feedlot mortalities and additionally results in the subsequent loss of performance and health [27]. Moreover, BRD diagnosis is usually dependent on trained feedlot personnel and is commonly based on observed clinical signs (e.g., cattle depression, nasal discharge, ocular discharge, coughing, gaunt appearance, or inappetence). Additionally, treatment often consists of administering antimicrobials which may lead to the increase of antibiotic-resistance determinants [7, 28].

BRD is usually observed in cattle within four weeks of transportation to a feedlot [29]. There are multiple clinical signs of BRD, which vary greatly, depending on the phase and extent of the disease process. The general signs can include depression, inappetence, dullness and fever. Additionally, several respiratory signs, including ocular and nasal discharge, coughing, excessive salivation, and abnormal respiratory rate and rhythm, have been observed in BRD-diagnosed cattle [30]. However, this assessment has limited sensitivity and specificity which may result in unnecessary treatment and delayed or negative detection of BRD in truly sick animals [31]. White and Renter [30] found that the sensitivity for BRD detection based on clinical signs observed by trained personnel was only 62%, indicating many BRD cases go undetected, or are not detected until the advanced disease stage when successful treatment is less likely [32]. Moreover, although the extent of lung lesions (e.g., pleural adhesions, collapse/consolidation, parenchymal fibrosis, abscesses, or emphysema) resulting from BRD is associated with the risk of mortality and retreatment, the lesions are frequently found at slaughter, often in calves in which BRD has never been detected [33, 34]. Overall, current methods for the early detection, prognosis and diagnosis of BRD still have low accuracy and therefore additional researches exploring BRD diagnostics are needed.

**Figure 1:**
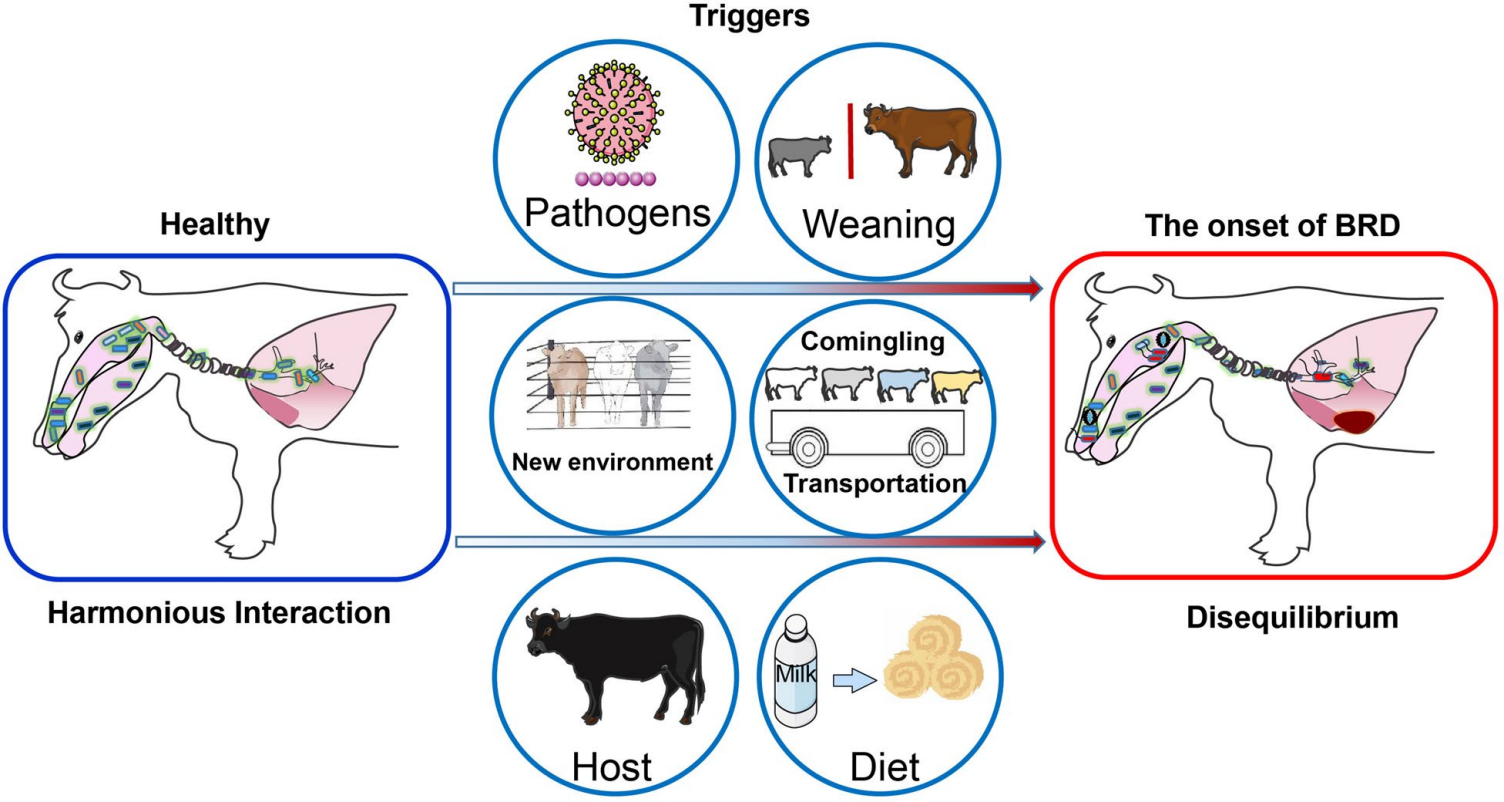
**Triggers affecting the healthy respiratory ecosystem and leading to the onset of bovine respiratory disease (BRD) in newly weaned beef cattle.** The bovine respiratory ecosystem has an increased risk of disequilibrium and subsequent BRD signs when the host is affected by pathogens, changes in the environment and managements (e.g., weaning, commingling, transportation, and dietary changes etc.).

The currently accepted theory regarding BRD pathogenesis is the complex synergistic interaction of bacteria and viruses under the influence of various stressors (i.e., weaning, comingling, transportation, and dietary changes) in addition to changes within the host and environment [35, 36] (Figure 1). A harmonious interaction between the host, properties of microbiome colonization and the local environment within the airways exists in healthy cattle. In contrast, a disequilibrium related to microbial dysbiosis, mucosal dysfunction as well as acute or chronic inflammation consequently generates an opportunity for the development of BRD [37]. So far, we know bacterial pathogen invasion produces the acute syndrome of BRD after the bovine respiratory system has been disturbed by factors such as viral infections, environmental changes and/or stress [38, 39]. There are multiple viral agents that can contribute to the development of BRD, including bovine viral diarrhea virus (BVDV), bovine respiratory syncytial virus (BRSV), bovine herpes virus 1 (BHV-1), and parainfluenza 3 virus (PI3V) [40]. Those viruses with stressors can lead to enhanced colonization and replication of bacterial pathogens and infect the lung subsequently. However, the incidences and abundances of these bacteria identified as BRD pathogens, which may be commensal organisms in healthy animals, do not correlate well with the occurrence of clinical BRD. For example, a high abundance of *Mycoplasma bovis* has been observed both in healthy steers and those diagnosed with BRD [41, 42]. Since knowledge gaps remain regarding BRD pathogenesis, a review of current research on community structure and composition of the microbiota of the bovine respiratory tract would allow for a better understanding of the pathobiology of BRD and emphasize an important direction for future research.

## Techniques used in the studies of the respiratory microbiome

Most BRD microbiology studies have been conducted using the nasopharyngeal swab (NPS) sampling approach [22]. Thus, sampling other niches within the respiratory tract is needed to better understand the cattle respiratory microbiome and BRD pathogenesis. This manuscript will describe the methods of sample collection in relation to the specific anatomical structures of the airway. The key points focus on subsequent measurements and data analysis of sequencing since most of the studies analyzed used the 16S rRNA sequencing technique.

### Sampling techniques for the bovine respiratory microbiome

For sample collection, the literature reports various methods when comparing the URT and the LRT. Typically, a sterile swabbing approach is common for the collection of microbiome samples from the URT. There are many types of swabs such as those with cotton or polyester ends with various transport medium available depending on the subsequent diagnostics employed. In several previous cattle studies, researchers used short cotton swabs (17 cm length) for nasal sampling, and longer double-guarded polystyrene cotton swabs (84 cm length) for nasopharyngeal samples [1, 22, 43]. Common approaches for the collection of LRT samples are mainly using trans-tracheal wash (TTW) and bronchoalveolar lavage (BAL) techniques [1, 43, 44]. TTW utilizes the insertion of a catheter into the trachea of subjects to collect fluids samples from the LRT to provide molecular evidence about the LRT for cytologic and culture analysis. BAL, as a minimally invasive medical procedure, employs a broncho-alveolar lavage tube passed through the nares into the lungs for sampling of the lower airways [45]. It is not well documented whether TTW or BAL is the best approach to investigate the LRT microbiome in cattle. In previous studies, TTW and BAL were able to determine the bacterial pathogens including *Mcoplasma* and *Mannheimia* in the lungs of calves acutely affected with BRD [1, 43], and *Mycoplasma spp.* and other bacteria have been isolated from BAL samples from pneumonia calves [46, 47]. Although some concerns remain regarding the risk of nasal and nasopharyngeal contamination, BAL has become more widely used for bacterial diagnosis in pneumonia due to its advantages of being minimally invasive and useful for cytology [45, 48, 49]. In human studies, the contamination of the BAL microbiome from the URT is considered minimal and could be ignored when using guarded BAL [12, 50, 51]. In addition to the common URT and LRT sampling methods described, some researchers have collected lung tissue post-slaughter for microbiome analysis [52]. However, the value of these samples is limited due to the cost of euthanasia, lack of slaughter facilities appropriate for the sampling of lung tissue and lung traceability. Overall, the most useful approach to sample the airway will be dependent on research goals, the diagnostics utilized, the species being studied, and the category and the severity of the disease course [53].

### Sequencing and bioinformatics for the bovine respiratory microbiome

Previous studies have analyzed the respiratory microbiota or pathogens using culture-dependent techniques [54–56]. However, these techniques only enable the detection of a small fraction of the microbiota. Also, various molecular techniques have been used to quantify specific microbes within the respiratory microbial community, including immunohistochemistry [57] and real-time PCR [58]. The improved availability of NGS techniques has allowed us to more broadly and specifically study the microbiome. Due to the low biomass of specimens in the LRT, the lungs have traditionally been considered a minimal source of bacteria when using culture-dependent or molecular techniques in the past [12, 15]. However, recent studies using NGS techniques have shown the complex microbial composition and found the common bacterial pathogens for BRD in the lungs of both healthy and sick cattle [1, 16, 23, 43, 52]. With the improvement of techniques, nanopore sequencing and multiple omics [i.e., metagenomics (the study of a collection of genomes and genes from the members of a microbiota by shotgun sequencing), metaproteomics, metatranscriptomics and metabolomics] are becoming more effective to investigate the composition and functions of the respiratory microbiome [59, 60]. In cattle, the bacterial community and BRD-associated pathogens were found when using metagenomics to measure all the genes of all the microbes in an environment [20, 25, 61–63], but still no reports using other omics were found. However, due to many factors such as cost, technique error, and time of analysis etc., NGS is still the most popular approach [64].

Big data analysis post-NGS is another key factor in respiratory microbiome analysis. After obtaining sequencing data from Illumina MiSeq /HiSeq or 454 pyrosequencing platforms [65–68], the quality control steps are carried out to remove low-quality reads with sequencing errors while the remaining high-quality sequences are classified to the genus- or species- level based on available databases such as RDP [69], Greengenes [70], NCBI [71] or BRD niche specific database [72]. Of note, it is critical to include negative (i.e., blanks during DNA extraction and PCR reactions) and positive controls (i.e., mock communities with known bacterial taxa) to rule out any environmental contaminations, especially when analyzing low biomass lung samples. The software, such as quantitative insights into microbial ecology (QIIME) and mothur, have been commonly used for 16S rRNA sequencing data analysis, including quality control, bacterial classification, and downstream analysis [73–75]. Then, basic analyses, such as community measures of alpha diversity (the diversity within a particular ecosystem, including richness and evenness) and beta diversity (comparison of diversity or the extent of changes between ecosystems) for overall community structure as well as major microbiota composition, are performed and reported.

### Statistics for the bovine respiratory microbiome

The interactions among microbes within a community are essential to study the bovine respiratory microbiome and its association with BRD. Network theory can investigate and display the complex interactions of microbiota in a single network. A wide range of methods has been used to build ecological networks regarding microbiome data [76]. Those methods vary in their accuracy, efficiency, speed and computational requirements, as well as the span from simple measures of pairwise Spearman or Pearson correlations, to more complex multiple regression and even Gaussian graphical models. A study using co-occurrence analysis by calculating all coefficients of Spearman’s rank correlation found “core” community structures formed between bacteria in both healthy and diseased human airways [77]. Only one study using network analysis related to BRD was found based on our knowledge and this research distinguished the bacteria associated with BRD using network analysis [78]. Network analysis should be used broadly in BRD researches, which could allow us to understand the microbial interplays and find the potential probiotics.

Identification of bacterial taxa related to BRD is of particular interest to many scientists. Many machine learning techniques that allow algorithm to be more accurate at predicting outcomes could be used to identify specific bacteria deferentially represented between healthy versus BRD calves, including Random Forest [79], Support Vector Machine (SVM) [80], Linear discriminant analysis Effect Size (LEfSe) [81], etc. For example, Random Forest has been used as a robust machine learning technique to identify microbial biomarkers related to different human and animal diseases [82, 83]. It can deal with binary, categorical and continuous variables, and works well with unbalanced data sets. Although machine learning needs more computational power and resource, it is not a big problem with the development of computer science. Area-under-ROC curve (AUC) of the Random Forest (AUCRF), that has been previously published [84], has higher accuracy for feature prediction. A recent study used machine learning to predict viral-induced BRD with high accuracy based on public datasets [85]. Moreover, regression-based Random Forest models could be developed to identify bacterial taxa associated with continuous variables such as body weight and temperature using an updated method. This exciting area of research is continually developing as new techniques and modified techniques are being explored and will ultimately be key to improving our recognition of bacterial pathogens.

Another important ecological question involves the spatial dynamics (from the upper to the lower airway) of the respiratory microbiome in the context of BRD. In humans, the URT microbiota disperses and colonizes the lung via respiration and microaspiration [86], and the island model has been used to detect microbial movement/dispersion from the source to sink environment community [87]. The island model considers source ecological communities (URT) as dynamic assemblages of microbes whose existence, deficiency and relative abundances in the sink (LRT) environment are affected by random dispersion, speciation, elimination, and stochastic birth and death events [88]. Some alternate models, such as the neutral model and source tracker, are useful to characterize the spatial movement of the respiratory microbiota as well. Source tracker can determine the contribution of microbiota from one or more sources to a particular sink using the Bayesian algorithm [89]. The neutral model assesses the microbial drift from one source to one sink [86]. Due to the complex ecosystems of bovine respiratory tracts, these models may not perfectly fit the dispersals of bovine respiratory microbiota. However, the outputs of these models should clarify the activities of BRD pathogens somehow. Unfortunately, there are limited BRD studies to measure the spatial dynamics of bovine respiratory microbiota [90]. In future BRD studies, researchers need to determine the microbial movements from the URT to the lung in both healthy and sick calves using these developing models, which may be helpful for the understanding of BRD pathogenesis.

Several statistical models have been developed and could be applied to examine the associations between the bovine respiratory microbiome, host phenotypes (e.g., body weight, BRD) and genotypes, diets and environment. For example, permutational multivariate analysis of variance (PERMANOVA) was used to screen for the factors influencing bovine respiratory microbiota [90]. Correspondingly, a study used bovine respiratory pathogens to predict the clinical outcome using a univariate logistic regression model [21]. Additionally, some analyses, such as Procrustes and multiple co-inertia, are approaches to integrate multi-omics datasets [91], and could be potentially applied to study bacterial-host interactions in BRD cases. These algorithms could allow us to explore the mechanisms of BRD pathogenesis and better identify the factors leading to BRD if they will be used in future studies of the bovine respiratory microbiome.

## Biogeography of the bovine respiratory tract and microbiota

**Figure 2:**
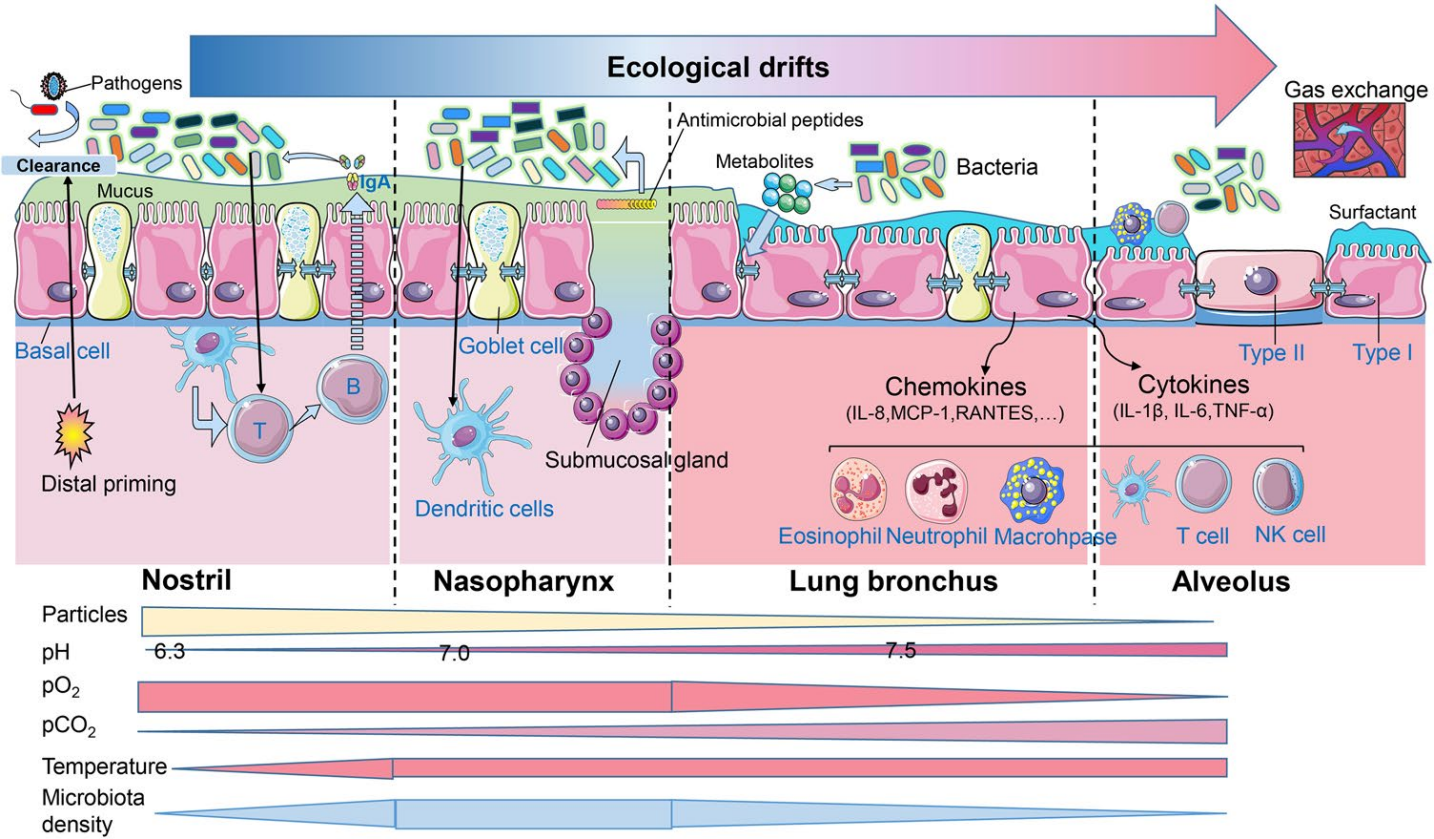
**The harmonious interaction of the respiratory ecosystem in healthy cattle.** The complex respiratory ecosystem in healthy cattle is harmonious and contains niche specific environmental properties, microbiota immigrations and host-microbiota interactions, which could resist pathogen colonization to some extent. The gradients of physiological features are along the respiratory tract and move from the nasal cavity to the nasopharynx to the trachea and terminate in the lungs. Lower temperature is reported in the nostrils while the lungs reach body temperature. The partial pressures of airway oxygen (pO2) and carbon dioxide (pCO2) have opposing gradients that are regulated by ventilation and gas exchange at the epithelial surface of the airway [11]. Concerning respiration and microaspiration, micro-particles from the external environment enter the upper respiratory tract (URT) and move to the lungs. Equilibrium is achieved when the host and the respiratory microbiota maintain harmonious interaction [15, 37, 108, 109].

The URT includes the nostrils, the tonsils, and the nasopharynx, whereas the LRT includes the larynx, trachea, tracheal bronchus, tracheal bifurcation, bronchi, bronchioles, alveolar ducts, alveolar sacs, and alveoli. Considering cattle, the tracheal bronchus specifically arises cranial to the tracheal bifurcation. The primary physiological function of the respiratory tract is to conduct gas exchange (inhalation of oxygen for the exchange and exhalation of carbon dioxide) from the blood. To achieve this, the respiratory tract must warm, filter, and humidify inhaled air and, in doing so, prevent the formation of poisonous or infectious agents that may have access to the respiratory system and threaten function and health. Thus, the pH and temperature gradually increase along the respiratory tract, while the partial pressures of airway oxygen (pO2) and carbon dioxide (pCO2) have opposing gradients that are regulated by environmental air circumstances and the ability of gas exchange at the surface of the lungs (Figure 2). The respiratory microbiota colonizes along the URT and further disperses into the LRT due to the respiratory tract’s anatomical connection with the external environment and direct dispersal along mucosal surfaces, where the microbiota lives. However, the niche-specific microbial communities along the respiratory tract are selectively grown due to the niche physiological parameters (e.g., temperature, pH, ventilation, etc.) that are present within the bovine respiratory tract [11]. In one study, the existence of *M. haemolytica* in the cattle nasal cavity affected its prevalence in the trachea, although their abundances in both sites were not well correlated [92]. Therefore, understanding the biogeography of the respiratory microbiome provides insights into the complexity of the respiratory ecosystem and BRD.

The URT contains several diverse anatomical zones that have different physiological conditions and a greater prevalence to generate contact with the external environment (e.g., diet, water, feces, urine) and other cattle. The nasal cavity, most rostral to the external environment, contains a skin-like, keratinized squamous epithelium. The genera associated with common BRD pathogens such as *Mycoplasma*, *Mannheimia,* and *Pasteurella* are observed in the nostrils of both healthy and BRD affected cattle [1]. Other dominant genera in the bovine nasal cavity, including *Psychrobacter*, *Aggregatibacter*, *Sphingomonas*, *Corynebacterium* and *Coprococcus*, are also reported [1, 41]. The microbial community of the nasopharynx, the region near the caudal aspect of the nose, has been widely investigated. The four genera associated with BRD pathogens, including *Mycoplasma*, *Mannheimia*, *Histophilus* and *Pasteurella*, have also been observed in nasopharyngeal samples from both healthy and BRD-affected animals [19, 22, 24]. Other dominant genera include *Pseudomonas*, *Psychrobacter*, *Actinobacillus*, *Clostridium*, *Acinetobacter*, *Bacillus*, *Proteus*, *Bifidobacterium*, *Rathayibacter*, *Cellulomonadaceae*, *Corynebacterium*, *Jeotgalicoccus*, and *Planomicrobium* [22, 23, 25, 93, 94]. Temporal changes of the nasopharyngeal microbiota have also been reported. A previous study in cattle confirmed that the nasopharyngeal microbiota changed significantly within several days of arrival to the feedlot, resulting in greater microbial diversity and richness [93]. *Pasteurella haemolytica* was identified in the tonsils of calves [95]. In addition, the microbiome in the oral cavity and oropharynx is also of interest since *Pasteurellaceae* associated with the commonly isolated BRD pathogens was detected in the oral cavity of calves [96]. Considering cattle often lick their noses and can actually reach farther into their nostrils than other species, it is not surprising that there would be similarities between the microbes found in the oral cavity and oral pharynx and the URT. However, studies are currently limited regarding the characterization of the oral microbiome in bovines. The first study to investigate the oral microbiomes of cattle with bovine periodontitis found that the most prevalent bacterial microbiota in cattle considered healthy were *Pseudomonas*, *Burkholderia* and *Actinobacteria*, whereas *Prevotella*, *Fusobacterium* and *Porphyromonas* were significantly reported in diseased subjects [97]. A recent study found *Streptococcus* was the predominant bacteria on the mouth floor, while *Streptococcus*, *Bibersteinia* and *Mycoplasma* were found in the oropharyngeal community in healthy calves [16]. Currently, no published studies of the microbiota found in the oropharynx of BRD-affected cattle exist. The oral and oropharyngeal microbiome may be a new direction for BRD microbiome research, since overlaps of *Mycoplasma* between the oropharynx and the lungs in cattle have been reported [16].

The LRT is comprised of trachea, tracheal bronchi, bronchioles, and alveoli. Passageways entering the lungs distal to the tracheal bronchus and bifurcation contain bronchi (primary, secondary, and tertiary), bronchioles, alveolar ducts and sacs along the respiratory tree [98, 99]. Until now, little to no bovine research has been conducted that separates the LRT into the trachea and lung to investigate the LRT microbiome, likely due to the supported evidence showing their similarity in the identification of BRD pathogens [44]. However, the abundances of non-dominant abundance bacteria between the trachea and the lung were different [16], and they may have functions in BRD pathogenesis that we may not yet understand. There are some studies that have started to investigate the LRT microbial composition and structure in cattle with different sampling techniques. In the clinically healthy bovine LRT, the genera *Mycoplasma*, *Moraxella*, *Pasteurella*, *Mannheimia*, *Bacteroides* and *Clostridium* were observed in both TTA and BAL samples [1, 20, 43]. Other genera such as *Bibersteinia* and *Prevotella* were also observed in the bovine lung [43].

Microbial movement or dispersion within the respiratory tract is another new research direction since it could potentially explain the contribution of the URT microbiota to the lung microbiota. One study concluded that nasopharyngeal microbiota may serve as the primary source for the lungs in healthy calves since the nasopharyngeal region shared similar bacterial composition with the lungs compared to other sampling niches [16]. Similarly, bacterial overlaps between the URT and LRT in cattle have also been described in additional studies [1, 23, 43]. However, in cattle, no studies have yet evaluated the dispersion of the respiratory microbiota with effective statistical models. In healthy subjects, microbiota enter the lungs through an active and continuous process by inhalation of air, direct mucosal dispersal and microaspiration from the URT (Figure 2) [50]. In healthy humans, the adapted island model hypothesizes that the lung microbiome and its growth rate are more affected by microbial immigration and elimination processes than by the effects of the local or lung growth environments [100]. The composition of a healthy lung’s microbiome fit a neutral model when using oral microbiota as a source, which meant the oral microbiota is one of the major sources contributing to the human lung microbiome [51, 86]. Moreover, Venkataraman et al. [86] found that 75% of the oral OTUs were neutrally distributed bacteria from the upper gastrointestinal tract in humans. Shared microbiotas between the oropharynx and lung, such as *Mycoplasma* and *Moraxella*, have been found in healthy cattle [16]. Therefore, especially for ruminants, investigation of microbial movement within the airway affected by external sources is complex yet relevant. Since the specific ruminating activity of cattle causes rumen content and rumen microbiota entry to the oral cavity and oropharynx frequently, it is expected that this physiological activity would influence lung microbiota [101], perhaps even more so than it might in monogastric. The shifts in rumen microbiota affected by dietary changes and age may also contribute to the structure of the respiratory microbiome. A previous report found that weaned calves which consumed selenium-biofortified alfalfa hay for nine weeks resulted in favorably reformed microbial communities in the nostrils [102]. Simultaneously, the communication between cattle and the environment, as well as with each other, could also serve as a significant contributor to the URT microbiota and, subsequently, the lung microbiota. Cattle, in particular young calves and dairy breeds (like Holsteins and Jerseys), tend to investigate their environment with their mouths. They lick, suckle, and mouth things in the environment (and each other) frequently, which suggests the environment could serve as an important source for both the oral and URT microbiota. Also, social grooming through licking the head and neck of each other is an important behavioral feature that may additionally influence oral and URT microbiota. Another potential source to consider is the direct inhalation of air in different weather conditions as survivability of microorganisms in the air can be influenced by temperature, humidity, UV light, etc. [11, 103]. These dynamic movements of microbiota are relevant for our understanding of pathogenesis in pulmonary health and disease [104]. For example, altered respiratory rate and effort in a diseased animal may alter the impact that direct inhalation of air might have on the microbiome. Additionally, diseased animals often have altered eating habits, such as inappetence, are sometimes offered diets higher in roughages such as hay, and may also receive antimicrobials in their diet, which may also impact the respiratory microbiome. Moreover, there are multiple factors that affect the URT microbiota and eventual microbial shifts in newly weaned calves, including antibiotics, external environments, stress, and host immunity [11, 15, 105, 106]. Recently, a study confirmed that a single injection of antibiotic including oxytetracycline and tulathromycin led to changes in the nasopharyngeal and fecal microbiota, and increased the relative abundance of several antibiotic resistance genes in these two communities later [28]. Therefore, investigation of microbiota dispersion through the URT or the oral cavity or oropharyngeal region needs to consider all these potential factors that could influence the bovine respiratory ecosystem.

## Association between the respiratory microbiome and BRD

Disease is one of the main factors influencing the respiratory microbiome. Many studies have confirmed that the alteration of the respiratory microbiota can be observed in calves with clinical BRD. For example, the nasopharyngeal microbiota in BRD-affected feedlot calves was distinct from pen-matched healthy controls [22, 103], a distinct longitudinal shift of microbial composition of the nasopharynx from feedlot arrival to when BRD was diagnosed was observed in another study [90, 107], and bacterial families associated with BRD, including *Mycoplasma* and *Pasteurellaceae*, had greater abundances and frequencies in lung tissue samples from calves with BRD signs [52]. Although these reports support the hypothesis that microbial dysbiosis is associated with BRD, we still do not know the accuracy of the association between the microbiome and BRD despite the microbial changes found. A key unknown factor is whether the pathogens and the rest of the microbiota are the inducers of inflammation and onset of BRD, or whether host inflammation or other changes in the respiratory immune system leads to the alteration of microbial structure by selectively overgrowing pathogenic microbes thriving in a more inflammatory milieu [37]. Therefore, integration of the mechanism of microbial dysbiosis or host immunity influenced by causal agents is important for clarity regarding the association of the respiratory microbiome and BRD.

### The microbial ecosystems in healthy bovine respiratory tracts

Understanding the physiological functions of the healthy airway and resident microbial colonization help us further elucidate BRD pathogenesis. In healthy cattle, a mucosal layer covers the respiratory tract and provides immune and physical protection for maintaining homeostasis under the interactions of the host, microbiotas and the external environments [15, 37, 108, 109] (Figure 2). Mucus consists of a complex array of antimicrobial peptides, immunoglobulins, glycoproteins, mucins, polysaccharides, ions, cells, and bacteria which work to maintain harmonizability [15, 109]. In addition, mucus in the URT offers protective barriers against pathogens and toxins from the external environment [110]. Dynamic mucociliary escalator transport works to shift mucus and the small particles it traps (dust, infectious agents, bacteria, etc.) toward the nasopharynx or oropharynx to be swallowed, which helps prevent foreign material from entering the lungs during breathing [111]. The nostrils, nasopharynx and trachea are lined with respiratory epithelium encompassing goblet cells containing pseudostratified columnar epithelial cells for mucous production [112]. Epithelium in the bronchioles shifts gradually toward a cuboidal epithelium with some cilia and club cells which generate glycosaminoglycans and secretory proteins to maintain normal lung physiology and host defense. Epithelial cell surfaces of the alveoli consist of two types: type I cells that regulate gas exchange processes and barrier function, and type II alveolar cells producing lipid-rich surfactants with the ability to prevent bacterial growth. These two epithelial cell types provide the mechanical defenses based on antimicrobial peptides whose secretion increases during the process of inflammation due to their activation by dendritic cells and macrophages [113]. They might also yield cytokines and chemokines that employ and trigger immune cells in infected or damaged areas of the respiratory tract [114]. Surfactant proteins A and D in type II and club cells (formerly named clara cells) potentially have antimicrobial and immunomodulatory roles by binding and inactivating microbial agents [109]. Moreover, the airway microbiota and their metabolites can cooperate with the host to protect against pathogen invasion/overproduction. Steed et al. [115] reported a microbially produced metabolite in humans (desaminotyrosine) that can protect the host by amplifying type I IFN signaling. Other factors, such as the secretion of the ion chloride as well as sodium uptake, are essential to maintaining normal regulation of mucus production [116]. Altogether, the microbiome adapts to a state of microbial symbiosis and homeostasis with the host mucosal surface and immune system. This complex interaction of factors requires additional researches in order to fully understand how different factors (environmental, host, pathogen) can impact this delicate balance.

### The microbial ecosystems in the respiratory tracts of BRD calves

Newly weaned beef calves experience numerous stressors that can cause dysbiosis of the respiratory ecosystem and result in subsequent BRD infection [22, 26, 102, 117, 118]. At feedlot arrival, the homeostasis of microbial communities in healthy cattle prevents pathogens from establishing infection on mucosal surfaces through the consumption of all presented nutrients, the adjustment of the local niche environments and microbial composition, the occupancy of receptor sites, the clustering of antimicrobial molecule construction, and the regulation of mucosal inflammation [11, 15, 109]. A previous study confirmed that the URT microbiota in healthy feedlot cattle rapidly changed from weaning day to arrival at the feedlot (a period of 2 days including 10 h shipping and overnight comingling with other calves) and within 40 days after feedlot arrival [119], indicating that the respiratory ecosystem responded to new challenges in the feedlot. In a previous study, the serotype 2 of *Mannheimia haemolytica* was more abundant in the nasal cavity before stress (before weaning and shipping to feedlot), while serotype 1, commonly considered to be more pathogenic, was more frequently isolated after feedlot arrival [120]. Likewise, viral and bacterial pathogen invasion through susceptible host defenses leads to microbial dysbiosis, damage of airway tissue and the subsequent development of BRD after feedlot arrival [15]. The proliferation and movement of BRD pathogens within the bovine respiratory tract were still unclear. A previous hypothesis stated that initiation of dysbiosis is directed by the stress and colonization of pathogens into the URT, subsequent shifting of the URT microbiome structure and then proliferation and ultimately infection of the lungs [121]. However, the mechanism of infections in BRD lungs caused by bacterial pathogens is not understood since pathogens are found in healthy lungs in calves. The spatial dynamics of pathogens and their subsequent influences on the bovine respiratory microbiome are necessary to be investigated in future studies.

**Figure 3:**
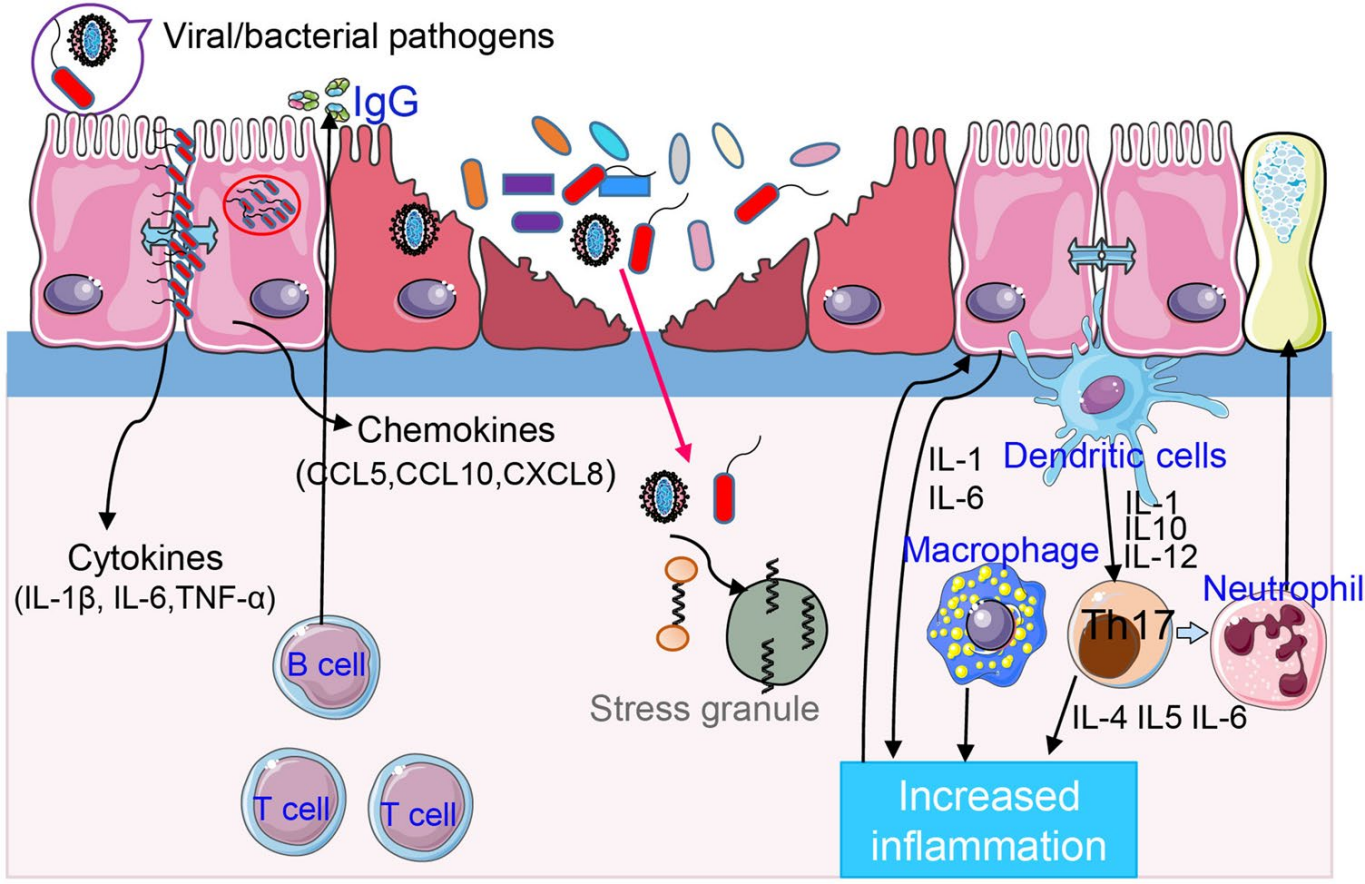
**Respiratory pathogen invasion and the host immune response.** Dysbiosis is developed by increased colonization of pathogens into the upper respiratory tract (URT), shifting the structure of the URT microbiome and then proliferating and infecting the lungs. In the pre-BRD state, the mucosal barrier’s function responds to dysbiosis of the bacterial community and the reproduction of pathogens across the airway epithelium by releasing chemokines and cytokines in mucus production and activates local immune cells [37]. The onset of BRD occurs when the unbalanced pre-BRD state suffers a decline into the clinical exacerbation state. Then, detectable damaged epithelium accompanied by a functional deficiency of the mucosal barrier is commonly found in the respiratory tracts of BRD calves.

While limited information for the colonization, dispersion and infection of BRD pathogens have been reported, our efforts in preventing and predicting BRD would be improved by increasing our understanding of the initial colonization of pathogens in the URT. The pattern-recognition receptors in the URT that are expressed by the mucosal epithelium and immune cells (e.g., dendritic cells, macrophages, and neutrophils) can recognize pathogens or other noxious substances and then provide signals to regulate acquired immune responses [110]. Increasing pathogen loads generate the URT immune responses, alteration of the niche environment, and subsequent disequilibrium. Under the initial stage of dysbiosis representing an increase in pathogens and host inflammation, the host is considered to be at the “pre-BRD” state in which microbial communities are unstable and easily breach susceptible host defenses. The mucosal barrier function responds to respiratory ecosystem dysbiosis and pathogen invasion by secreting signaling molecules (e.g., inflammatory cytokines and chemokines) in mucus production, and the stimulation of an immune response in local niches [15]. This pre-BRD state can be considered a reversible phase. To resist pathogen invasion, protect the host, and reestablish barrier function, inflammatory events (including IgA production, immune cell recruitment) are activated until risk signals vanish, and reestablishment of the damaged area can begin to occur [37]. Alternatively, the onset of BRD occurs when cattle in the unstable pre-BRD state are subjected to the continuous increase of pathogens. Then, detectable mucosal damage accompanied by a deficiency of the mucosal barrier is usually found in the respiratory tracts of BRD-affected calves (Figure 3).

Citing *M. haemolytica* as an example, it is a commensal resident in the URT of healthy calves. However, a sudden explosive proliferation associated with stress and viral infection occurs in the URT of susceptible animals [122]. One specific serotype of *M. haemolytica* (serotype 1) adheres to and colonizes bovine bronchial epithelial cells, and subsequently forms foci of infection through damaging tight-junction integrity, transcytosis and rapidly replicating intracellularly [123]. *M. haemolytica* produces leukotoxin and lipopolysaccharide which are two important virulence factors that contribute to *M. haemolytica’s* pathogenicity within the respiratory tract [124]. During the invasion process, *M. haemolytica* stimulates host epithelial cells producing proinflammatory mediators including cytokines (TNF-α, IL-6 and IL-1β) as well as chemokines (CXCL8). The generation of proinflammatory mediators and the release of lipopolysaccharide and leukotoxin together affect neutrophil movement into the lungs, as they are the primary cause of damaged respiratory tissue associated with BRD [125–127]. Then, the BRD pathogens, such as *M. haemolytica*, could attack differentiated bovine bronchial epithelial cells through cytopempsis, and replicate quickly in cells prior to BRD triggering subsequent widespread cellular injury and lung lesions [123, 125].

### The remained questions for the association between BRD and microbiota

Until recently, the actual mechanism of epithelial damage in BRD cases has not been clear due to the complex interaction of multiple viral and opportunistic bacterial pathogens. Although the process of bovine respiratory viruses cooperating with bacterial pathogens is not clear, in humans, the summarizations of how respiratory viruses interacted with bacteria generating damage peripheral to the bronchi and bronchioles are reported [128]. The presence of viruses in the bovine respiratory tract may also alter the microenvironment of mucosal surfaces and bacterial structure, leading to reduced mucociliary function and cilia damage. Additionally, viral infection could reduce the concentration of antimicrobial peptides, bacterial adherence and invasion by adjusting the host immune system [129]. Segal et al. [130] observed that enhanced expression of inflammatory cytokines (Th17 immune activation) is associated with bacteria in the human LRT, which indicates that the respiratory microbiota could regulate the inflammatory condition at the mucosal surface in humans. Host defense and clearance of viral infections are generally facilitated by an equilibrium of the humoral immune response to neutralize antibodies and the T cell-mediated immune response. Undeniably, viruses in the bovine airway may injure ciliated cells and even the epithelial layer by disturbing cellular tasks and killing infected epithelial cells, decreasing mucociliary transport and consequently exposing the respiratory tracts basement membrane [15, 19]. Thus, an altered niche environment could lead to secondary infections by opportunistic bacterial pathogens following a host immune response by facilitating adhesion and colonization of bacterial pathogens [131, 132].

There are additional knowledge gaps regarding how the microbiota, including commensal bacterial and pathogens, move within the airway of both healthy and BRD calves. Achievements in understanding microbial shifts within respiratory tracts in other species provide alternative statistical techniques to study microbial drift associated with BRD. In humans, the neutral model can estimate microbial drifts from the URT to the lungs successfully in healthy individuals, but fails in patients with cystic fibrosis [51, 86]. In future studies, the microbial drifts in the bovine respiratory tract need to be investigated using similar statistical approaches by considering the specific factors influencing the lung microbiome in cattle, to provide more insights into the pathogenesis of BRD.

## Conclusions and future perspectives

BRD is a multi-factorial disease and the pathogenesis of BRD is complex and not fully understood. Although the rapid development of sequencing techniques and machine learning or data science help us to better characterize the biogeography of microbial communities in the respiratory tracts of healthy cattle and those affected by BRD, there remain many knowledge gaps. The complexity of the respiratory ecological system limits our understanding of mechanisms such as microbial colonization and host-microbiota interactions, and drift between biogeographical locations. Suitable techniques for sampling niches and data analyses should be considered and optimized based on research goals. Many studies have analyzed the differences of the respiratory microbial composition in healthy or BRD affected cattle in different niche locations.

However, little is known about the dynamic microbial movements within the airway [90]. Moreover, characterizing the function of the respiratory microbiome is one of the next steps and will be an important component in advancing our understanding of pathogenesis. Additionally, advanced multi-omics techniques (i.e., metagenomics, metaproteomics) will provide key insights into the respiratory microbiota and its interaction with epithelial cells for maintenance of homeostasis and dysbiosis associated with BRD development. Future studies concerning BRD and the bovine respiratory microbiome should also consider greater exploration of the interactions between viral and bacterial communities, and the association of microbial dispersion and BRD severity, to determine which factors (especially stress) contribute to pathogen colonization and proliferation. Simultaneously, the bovine respiratory microbiome data should be integrated with host phenotypic data to elucidate the impact on BRD pathogenesis.

Future studies investigating the temporal and spatial dynamics of the bovine respiratory microbiome using multi-omics approaches not only improve our understanding of the pathogenicity of BRD but also provide novel biomarkers for the accurate prognosis and diagnosis of this disease. For example, a panel of biomarkers including bacterial taxa, metabolites, and gene transcripts generated from nasal swabs collected from healthy calves and those with BRD could be developed by machine learning techniques such as Random Forest to predict and diagnose BRD if the nasal microbiome is representative of the bovine respiratory microbiome (at least the upper respiratory system). Advanced tools in bioinformatics should be developed to integrate the high dimensional datasets generated from multi-omics studies using different platforms. Network analysis of the lower respiratory microbiome can reveal bacterial interactions between the opportunistic pathogens and other bacteria. The bacterial taxa that had antagonistic interactions with these pathogens could be further investigated and used as potential probiotics to kill these pathogens. Overall, the deep and continued investigation of the bovine respiratory microbiome opens new possibilities for therapeutics and rapid detection approaches for BRD or other respiratory diseases.

## Abbreviations

BRD: bovine respiratory disease
NGS: next-generation sequencing
URT: upper respiratory tract
LRT: lower respiratory tract
BVDV: bovine viral diarrhea virus
BRSV: bovine respiratory syncytial virus
BHV-1: bovine herpes virus 1
PI3V: parainfluenza 3 virus
NPS: nasopharyngeal swab
TTW: trans-tracheal wash
BAL: bronchoalveolar lavage
QIIME: quantitative insights into microbial ecology
SVM: Support vector machine
LEfSe: Linear discriminant analysis Effect Size
AUCRF: Area-under-ROC curve (AUC) of the Random Forest
PERMANOVA: permutational multivariate analysis of variance
